# Influence of eating habits and alcohol consumption on the academic performance among a university population in the community of Madrid: A pilot study

**DOI:** 10.1016/j.heliyon.2021.e07186

**Published:** 2021-06-01

**Authors:** Miguel López-Moreno, Marta Garcés-Rimón, Marta Miguel, María Teresa Iglesias-López

**Affiliations:** a Institute of Food Science Research (CIAL; CSIC-UAM), Madrid, Spain; b Grupo de Investigación en Biotecnología Alimentaria, Universidad Francisco de Vitoria, 28223, Pozuelo de Alarcón, Madrid, Spain

**Keywords:** University students, Alcohol consumption, Community of Madrid, Academic performance, Dietary habits

## Abstract

**Objective:**

To evaluate the association between dietary habits and alcohol consumption on academic performance among university students.

**Design:**

Cross-sectional study.

**Setting:**

University located in Madrid, Spain.

**Participants:**

56 university students of nursing (45 women and 11 men). The study complies with the Helsinki Declaration and was approved by the University Ethics Committee (36/2018).

**Main outcome measure:**

Dietary intake and habits assessed with validated survey, alcohol consumption and academic performance.

**Analysis:**

Chi-square test, Student's t-test, ANOVA analysis, Mann-Whitney test, Kruskal-Wallis analysis and Shapiro-Wilk test.

**Results:**

The average daily energy intake of the students was 1918 ± 725 kcal and, on average, alcohol accounted for 6%. The increased energy contribution from alcohol was negatively correlated with body mass index (BMI). Moreover, an inverse association was also found in alcohol intake according to Body Mass Index (BMI) (p < 0.02). Students with failing grades (53.6%) reported a higher daily alcohol intake than those who passed (42.2 %) (32 g/day versus 24 g/day) (p = 0.043).

**Conclusions:**

Alcohol consumption is related to both poor academic performance as well as diminished quality of life. Thus, it is of vital importance to undertake awareness campaigns at various levels to dissuade alcohol consumption especially at early ages.

## 1 Introduction

A healthy lifestyle consists of a series of actions, habits and patterns of behaviour acquired at an early age and persisting, in the most of cases, into adulthood contributing to good health. However, university students often have a poor diet being the intake/food consumption of female students generally better than their male classmates (Sánchez-Ojeda et al., 2015). Furthermore, the lifestyle of young university students often includes the consumption of drugs, and, in particular the consumption of alcoholic beverages, especially spirits, in this group of population is very high and it is currently considered a social and a public health problem (Mekonen et al., 2017b).

In Spain, alcohol consumption among young people was generally associated with social events and nowadays the traditional pattern of consumption consist in heavy episodes of drinking (intake of large amounts of spirits in a short period of time) (Ministerio de Sanidad Servicios Sociales e Igualdad, 2017). In the Spanish population is estimated that 37.5% of young men have suffered from alcohol intoxication in the last month, while in the case of young women are 22.6% (Ministerio de Sanidad Servicios Sociales e Igualdad, 2017). In terms of nutritional value, alcohol it is not considered a nutrient because only provides empty calories, which appear to stimulate the appetite and thus may be considered as a risk factor in the development of metabolic disorders and abdominal obesity, between others (Traversy and Chaput, 2015). On the cognitive level, alcohol abuse has been associated with impaired memory and various cognitive disfunctions (Fuentes et al., 2020) (Salas-Gomez et al., 2016), as well as its direct relation on academic performance (Mekonen et al., 2017b). Additionally, in the majority of cases, the period of university studies supposes a significant change in the lifestyle of students, which may have negative implications in their eating habits (Zurita-Ortega et al., 2018). This situation increases the risk of overweight or obesity. In this context, spirits have been associated with weight increase (Lukasiewicz et al., 2005). Lean et al. (2018) observed that alcohol consumption and frequency of consumption were positively and inversely associated, respectively, with Body Mass Index (BMI) and waist circumference (WC) in 35837 Scottish adults. Breslow and Smothers (2005) found an inverse relationship between alcohol-drinking frequency and BMI, whereas those reporting greater alcohol consumption/day had higher BMI. Tolstrup et al. (2005) observed in men and women that drinking frequency was independently and inversely associated with high BMI, meaning that the lowest odds of being obese was observed among the most frequent drinkers. With regard anthropometric measures, was found that drinking frequency was inversely associated with large waist and directly associated with small hips (Tolstrup et al., 2005).

Numerous studies have shown the importance of a healthy food habits and lifestyle in the correct cognitive development and functionality in adolescents (Barchitta et al., 2019; Karande, 2019; Kim and Kang, 2017; Martin et al., 2018). Although in the general population alcohol consumption is more prevalent in men than in women, the prevalence of excessive high intake is similar in both sexes in university students (Dantzer et al., 2006). However, very few studies have evaluated the impact of certain lifestyle habits on the cognitive performance of university students. The brain of young adults is susceptible to the neurotoxic effects of alcohol binge drinking (López-Caneda et al., 2014). As pointed López-Caneda et al. (2013), an association between this pattern of alcohol consumption and neurofunctional effects have been observed. Moreover, risky alcohol consumption and heavy episodic drinking are predictor of poor academic achievement (Mota et al., 2010). University students are particularly at risk for these consumption patterns when living away from the family home. Different studies have addressed potential factors that contribute to hazardous drinking among university students (Colomer-Pérez et al., 2019; Flaudias et al., 2019; Mekonen et al., 2017a). Thus, the aim of the present study was to evaluate the prevalence and impact of alcohol consumption, eating habits and body weight on academic performance among nursing university students in the Community of Madrid.

## 2 Methods

### 2.1 Study design and participants

A cross-sectional study was conducted in students that are in the second year of a Degree in Nursing. Prior to the start of the study, the participants were informed of the purpose of the research and all signed an informed consent form. The study protocol and design were approved by the Ethics Committee of the University Francisco de Vitoria (36/2018), and it fully complied with the 1964 Helsinki Declaration and its later amendments. The evaluation protocols were provided physically, and the data was uploaded onto a database for analysis. The data were collected during scholar hours with any incidents and analyzed anonymously. The inclusion criteria were that they must be studying during the second year of Nursing Bachelor, with mean age between 18-25 years old. Students older than 25 years were excluded in this study. Individuals were excluded if they did not finish properly the questionnaires or had health problems (non-communicable diseases or contagious diseases).

### 2.2 Anthropometric measurements

Anthropometric measurements were carried out using calibrated digital scales SECA® (SECA Vogel & Halke, Hamburg) as well as portable stadiometers SECA® (SECA Vogel & Halke, Hamburg). Students’ weight was measured barefoot and wearing light clothes in kilograms, to the nearest 100-gram unit (0.1 kg), and stature was measured with the subject standing fully erect with feet together, head in the Frankfort plane, and arms hanging freely to the nearest millimetre (0.1 cm). Body mass index (BMI) was calculated using the formula weight (kg)/[height (m)2]. Subjects were classified as underweight (BMI <18.5 kg/m^2^), normal weight (BMI 18.5–24.9 kg/m^2^), overweight (BMI 25–29.9 kg/m^2^) and obese (BMI ≥30 kg/m^2^), based on the World Health Organization (WHO) criteria (WHO, 2000). Waist circumference was measured using an anthropometric measuring tape, at a horizontal plane midway between the lowest rib and the iliac crest. As cut-off points for risk identification for metabolic diseases waist-to-hip ratio >0.85 for female and >0.9 for male. The waist-hip ratio (WHR) was also calculated, following the formula: circumference of the waist (cm)/hip circumference (cm) (World Health Organization (WHO), 2011).

### 2.3 Questionnaires

**Figure 1:**
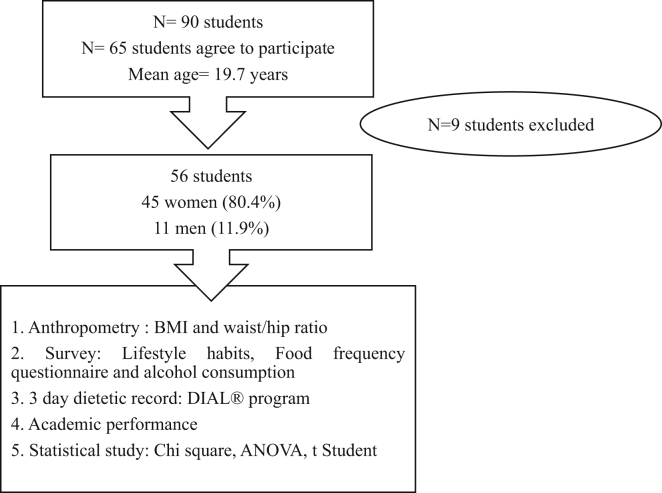
Study flow diagram.

The data collection process was carried out to determine both, the eating habits and alcohol consumption of the participants as well as their academic results (Figure 1). Data was collected through a face-to-face interview conducted by trained staff. Food and alcohol intake a was assessed using three-day food records (from Friday to Saturday), after receiving training in class time. Completed food records of the participants, were analysed using a computerized energy and nutrient analysis software (DIAL®) (Ortega RM, Andrés P, Requejo AM, Aparicio A, 2014). A survey was also conducted with certain lifestyle habits (Iglesias et al., 2015), including alcohol consumption (quantity, type and frequency) and sleep habits (hours of sleep per day). Healthy Eating Index (HEI) take into account the variety of food consumption (between ≤6 to ≥16 different foods) for 3 days and give a dietary rating of 0–100 points: assigning 0–16 points to cereals and legumes, 0–3 points to vegetables, 0–2 points to fruit, 0–2 points to dairy products, 0–2 points to meat, fish and eggs. Also, it takes into accounts %E fat (≥45%, ≤,30% E) %E SFA (>15%, <10% E), cholesterol (>450 mg/day, <300 mg/day), diet sodium (>4800 mg/day, < 2400 mg/day) and variety of food intake during 3 days (≤. 6 food intake/3 days, ≥ 16 food intake/3 days). The HEI score is: ≤ 50 inadequate, 50–70 acceptable-good and ≥70 very good-excellent (Ortega et al., 2014).

Data concerning of daily alcohol consumption (fermented or distilled) was calculated after the 3-days record. Individuals reported their intake of alcohol and grams and %E alcohol were calculated by means of the software program DIAL®.

Finally, academic performance was calculated as the average score of all subjects in the year 2018–19. The marks in Spain were categorized as: 1 (9–10) excellent; 2 (7–8) good; 3 (5) normal; 4 (4–1) worse. But to simplify, in this study we dichotomized the results into 1 (≥5) student with excellent to normal marks and 2 (<5) students who failed.

### 2.4 Statistical analysis

The statistical analysis was conducted using the IBM Statistical Package for Social Sciences (SPSS) version 22.0 (IBM, Chicago, IL, USA). for Windows (Figure 1). The continuous variables were expressed as the average and the standard deviation (SD) while categorical variables were expressed as percentages and frequencies. For the comparison of proportions, a Chi-square test with contingency tables was used. A Student's t-test and ANOVA analysis were performed to analyse the relation between quantitative variables and categorical variables with two levels or with more than two categorical variables, respectively.

When the continuous variables were not normally distributed, non-parametric methods were used such as the Mann-Whitney test in the case of two categorical groups or a Kruskal-Wallis analysis when there were more than two groups. Normality was confirmed using a Shapiro-Wilk test. The Spearman correlation coefficient was employed to assess the correlation between variables. The correlation coefficients were interpreted using the following thresholds: trivial (r < 0.1), small (0.1 < r < 0.3), moderate (0.3 < r < 0.5), large (0.5 < r < 0.7), very large (0.7 < r < 0.9) and extremely perfect (0 ≥ 0.9). The statistical significance threshold of p < 0.05 was used.

## 3 Results

The 90 students present in the class were invited to participate voluntary in the study and were informed of the procedure. Any possible doubts were addressed and the information sheet bout the study and the consent form were handed out. Of these 90 students,65 agreed to participate and 9 students were excluded due to missing data and. Finally, we obtain data of 56 nursing students all Caucasians: 45 women (80.4%) and 11 men (19.6%). The average age of both sexes was 19.7 ± 1.6 (Figure 1).

Table 1 shows anthropometric and dietary characteristics of the studied population, classified by gender. The analysis of the anthropometric variables showed a mean BMI of 23 ± 3.4 (24.6 ± 4.3 men vs 22.6 ± 3.1 women). In addition, 67.9% of students had a BMI classified as normal weight (18.6–24.9 kg/m^2^). Some 25% of participants were either overweight (BMI 25–29.9 kg/m^2^) and obese (BMI >30 kg/m^2^), with a higher percentage among men (45.5%) than women (17.7%). Significant differences were also found between gender with body weight (72.1 kg men vs. 62.5 kg women; t = 1.96), and gender with waist/hip ratio (0.88 men vs. 0.79 women; Z = -4.156) (p < 0.05).

**Table 1:** Anthropometric and dietetic characteristics of the university students in total and by gender.

	Women (n = 45) Average (SD)	Men (n = 11) Average (SD)	Total (n = 56) Average (SD)
Age	20 (2)	20 (1)	20 (2)
Weight (kg)	63 (11)	72 (12)	64 (11)
BMI (kg/m2)	22.6 (3.1)	24.6 (4.3)	23 (3.4)
WHR ratio	0.8 (0.1)	0.9 (0.1)	0.8 (0.1)
**Dietetic description**			
Energy intake (kcal)	1876 (711)	2088 (794)	1918 (725)
Proteins (%)	17.1	16.7	17
Fat (%)	34.8	34.1	34.6
CH (%)	38.7	44.1	39.7
Alcohol (%)	6.7	3.8	6.1
HEI score	55.4	58.5	56

In terms of diet, the average energy intake was 1918 ± 725 kcal/day, with carbohydrates accounting for the highest percentage of calories (39.7 ± 8.5%) compared to fat and protein (34.6 ± 9.8% and 17 ± 4.2%, respectively). A global evaluation of dietary habits showed that only 28.2% of students had an excellent HEI (36.4% men vs. 26.7% women), while 23.2% had an acceptable HEI (18.2% men vs. 24.4% women). In terms of hours of sleep, 42.9% of the students reported sleeping 7 h per day while 44.7% reported sleeping less than 6 h per day. However, no significant relation was found between hours of sleep and BMI or academic performance (p > 0.05).

Regarding alcohol consumption, participants reported an average intake of 12.9 ± 11.7 g/day, representing 6.1 ± 5.9% of their daily calorie intake. Regarding the preferred alcoholic beverages, beer was the most consumed by students (50%), followed by spirits (46.4%) and wine (33.9%). As for the frequency of consumption, the majority of students (83.9%) reported consuming alcohol at least once a week. In addition, 37.5% of the students reported they began consuming alcohol between the age of 16 and 17 years old. Surprisingly, students with a BMI below 18.5 were those having a higher alcohol intake (16.4 g/day), and significant differences were observed in alcohol consumption according to BMI (χ^2^ = 10.11, η^2^ = 0.20, p < 0.05). Moreover, an increased energy contribution from alcohol was negatively correlated with BMI (Spearmen's Rs = -0.33, p < 0.05) and this association also remained significant after adjusting for gender, exercise, total energy intake and HEI score (Table 2).

**Table 2:** Correlation analysis between energy intake from alcohol with anthropometric characteristics.

Variable	EIA		EIA adjusted	
	Rs	*p*-Value	Rs	p-Value
Age	-0.260	**0.046**	-0.268	**0.044**
Weight (kg)	-0.029	0.830	0.073	0.607
BMI (kg/m2)	-0.378	**0.040**	-0.303	**0.029**
WHR ratio	-0.313	**0.019**	-0.249	0.075

EIA, energy intake from alcohol; BMI, body mass index; WHR, Waist/Hip Ratio. HEI, Healthy eating index.

Note: Data presented as Spearman correlation coefficient. Statistical adjustment for gender, exercise, total energy intake and HEI score. Bold indicates statistical significance (*p* < 0.05).

The examination of the academic performance of the sample population showed that a high proportion of students (53.6%) resulted in failing grades, while only 10.7% resulted in very good or excellent scores. Stratified by gender, the failure rate was higher among women (57.8%) than men (36.4%), although these differences were not statistically significant (p > 0.05).

**Figure 2:**
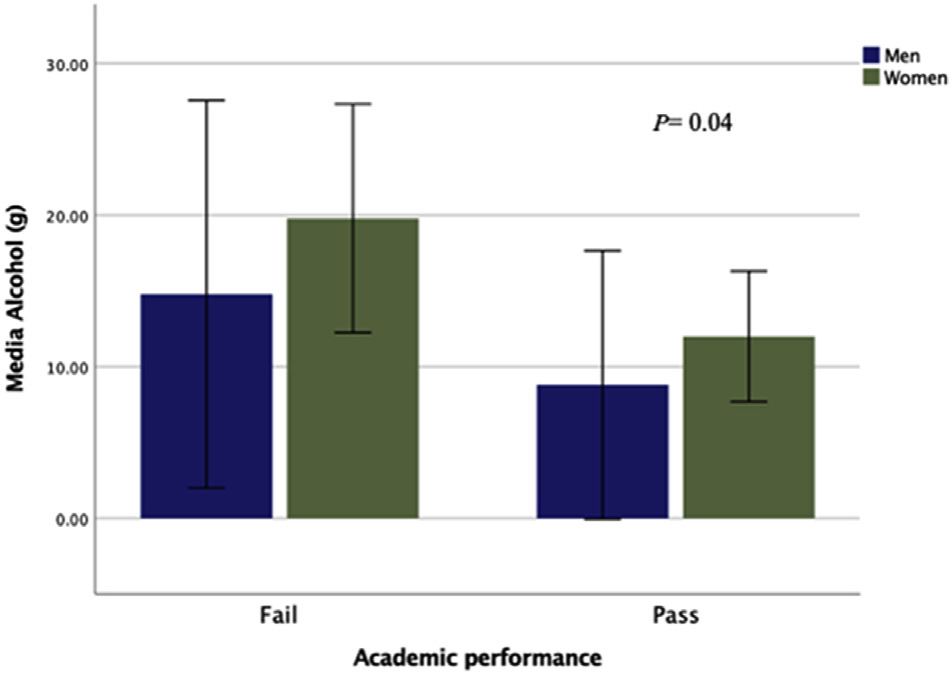
Academic performance according to alcohol intake by gender. Note: *P* values are based on group comparison using Mann-Whitney test.

**Figure 3:**
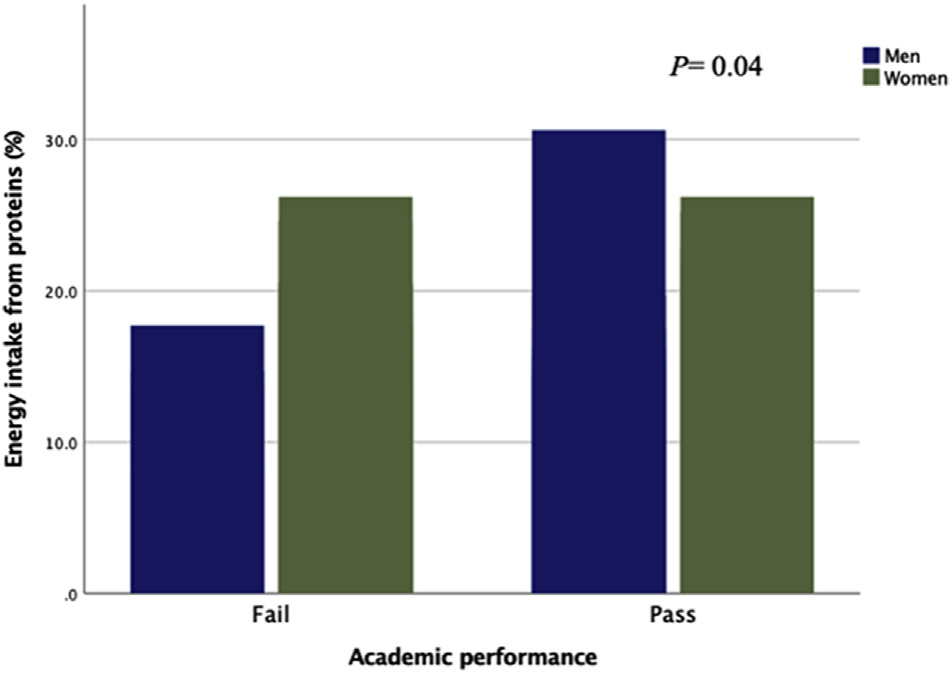
Academic performance according to percentage of energy intake from proteins by gender. Note: *P* values are based on group comparison using Mann-Whitney test.

Figure 2 shows academic performance according to alcohol consumption by sex. It was observed that students with failing grades had a significant higher rate of alcohol consumption (20.8 ± 12.9 g/day), compared to those with passing grades (11.1 ± 10.4 g/day). This relation was considered statistically significant (Z = -2.08, η^2^ = 0.96, p < 0.05). Differences were also found in academic performance with regards to rate of energy intake from proteins; passing students showed a significantly higher intake of proteins (Z = -2.22, η^2^ = 0.72, p < 0.05) (Figure 3). It was also observed that university students with poorer academic performance also showed a higher BMI. This association, however, was not statistically significant (p > 0.05).

## 4 Discussion

The aim of the study was to analyse the relation between dietary habits, alcohol consumption and academic performance of university students in the Community of Madrid. The results showed a relation between the BMI and certain dietary habits, as well as the negative impact of alcohol consumption on academic performance, while the intake of dietary protein among students showed the opposite trend.

In the present study, the mean dietary intake of total fat was higher than World Health Organization (WHO) healthy diet recommendation (not exceed 30% of total energy intake) (World Health Organization (WHO), 2020). In addition, a high proportion of students had a low-quality diet according to the HEI. This score have been associated with a lower risk of all-cause mortality, cancer, cardiovascular disease, diabetes and neurodegenerative disease (Schwingshackl et al., 2018). Similarly, almost half of the population studied did not meet the recommended sleep duration of at least 8 h of sleep per day (Chaput et al., 2018). This is especially relevant if we take into consideration that sleep disruption at different levels. A short duration of sleep affects the function of the hypothalamic-pituitary-adrenal axis causing an alteration in the regulation of hormones involved in appetite and energy expenditure (Lin et al., 2020). However, no association between sleep and body weight was observed in this study. These results are similar to those observed in a recent study of students in Morocco, which also found no significant differences between sleep duration and body weight (Benaich et al., 2020).

Considering that there is no alcohol consumption without risk to health, the findings in this university population are alarming (Amiri and Behnezhad, 2020; Bagnardi et al., 2015; Galán Labaca et al., 2020). On the other hand, there are few studies on substance misuse among nursing students in Spain. That is important because most nursing students will work as nurses or healthcare providers and this misuse could relapse in their patients. According to the ANIBES study of consumption habits, alcohol accounts for an average of 2.7% of the energy intake of the Spanish population. This is far below the data found in the university student population (6.1%). Additionally, it was found that the calories intake through alcoholic drinks were higher among women than men, in contrast to the findings of the ANIBES study (Nissensohn et al., 2016). However, our results are similar to those found in other university populations (El Ansari et al., 2013; Vorster et al., 2019). Some prior studies have suggested that in countries where drinking alcohol is considered is embedded in our current culture, university students show higher levels of alcohol consumption than non-student population (O’malley and Johnston, 2002).

Among adolescents, the characteristic habit of alcohol consumption is known as “binge drinking”. Previous studies have observed that 47.6% of Spanish university students had this massive intake of alcohol over a short period of time (Salas-Gomez et al., 2016). EDADES study found a prevalence of “binge drinking” among 30% of men and 16–20% in women among Spanish people from 20-29 years old (Observatorio Español de la Droga y las Toxicomanías, 2017). Other study of university students in Great Britain found that 21% showed possible signs of alcohol addiction according to the Alcohol Use Disorders Identification Test (AUDIT) (Heather et al., 2011). Although the parameters of this study do not included “binge drinking” specifically, we may pondered over this alcohol consumption is unfortunately prevalent among our sample population considering the high volumes of alcohol consumed. Furthermore, a significant relation was observed between alcohol consumption and academic performance (Figure 2). These findings are in line with the results of previous studies associating alcohol consumption with academic difficulties and less hours of study (Mekonen et al., 2017b)(El Ansari et al., 2013). This may be related to various social, psychological, economic and physiological aspects of the students. Alcohol consumption has also been associated with mental health problems, absenteeism, less motivation and academic integration (Iranpour and Nakhaee, 2019). However, research into the neuro-physiological impact of alcohol consumption have yielded disparate results. A study by Herrero-Montes et al. (2019) found no significant relation between cognitive performance and alcohol consumption among university students. Similar results have been observed by El Ansari and Salam (2020) in Finland. However, in this study academic performance was evaluated through the student's own subjective perception. On the other hand, various studies have found a significant relation between alcohol consumption and memory loss (Carbia et al., 2017) (Salas-Gomez et al., 2016), anxiety and depression (Moure-Rodríguez et al., 2016). This disparity in research results is primarily due to different criteria used to evaluate alcohol consumption and academic performance.

This study also found higher protein intake to be related to greater academic performance (Figure 3). The Mediterranean diet provides an optimum intake of proteins and appears to be the ideal diet to be encouraged among university students as it is associated with improved academic performance (Esteban-Cornejo et al., 2016; Gianfredi et al., 2018; Zurita-Ortega et al., 2018). Other studies have also found a relation between academic performance and healthy lifestyle habits (Hayek et al., 2020; Masoomi et al., 2020; Matthews et al., 2019). Adelantado-Renau et al. (2019) observed a positive correlation between adherence to the Mediterranean diet and academic performance among adolescents, mediated for quality of sleep. Additionally, a recent study found that higher levels of anxiety about exams and academic stress in general is related to poor adherence to the Mediterranean diet among university students (Trigueros et al., 2020). Furthermore, the present study also found a relation between the BMI and academic performance, although this association was not statistically significant and may be due to the limited size of the study sample. A recent study found a statistically significant correlation between BMI and academic performance (β = 0.41 for boys and β = 0.46 for girls, p < 0.001) (Ma et al., 2020). The impact of a high BMI on academic performance has previously been described by various authors who have associated a diet based on hyper-palatable foods (high in sugar and fat) with insulin resistance, oxidative stress and the low grade inflammation, which can ultimately have a deleterious effect on cognitive functions (Freeman et al., 2014; Kim and Kang, 2017). Additionally, it has been demonstrated that a high intake of highly processed foods at an early age may predict poor academic performance in later years (Anderson and Good, 2017; Purtell and Gershoff, 2015).

Additionally, some authors suggest that academic performance of adolescents may be influenced by eating disorders (Adelantado-Renau et al., 2018; Weider et al., 2014). Thus, this situation may be aggravated during their university studies, a period characterized by irregular eating habits, increased stress and insufficient sleep, as it was found in our study. The InSOMNIA study conducted on university students found a statistically significant correlation between different sleep disorders and the BMI (Gianfredi et al., 2018). Sleep deprivation, a characteristic of this population, can lead to alterations in the circadian rhythm of certain orexigenic and anorexigenic hormones which may induce hyperphagia leading to obesity (San-Cristobal et al., 2020).

The results of this study reveal an association between academic performance and dietary habits, especially with regards to habitual alcohol consumption among university students. However, certain limitations of this study have been identified, including the design of the study itself. As a cross-sectional study, the capacity to establish causal relationships is limited. This is a preliminary study based on a limited sample of only 56 university students, which conditions its external validity. The results should be confirmed by studies using a larger sample, over time. In our sample population, a large part of the participants were women (80.4%) and, thus, it is difficult to extrapolate the results to the male university population. Furthermore, it is highly probable that variables such as alcohol intake should be underreported, given that the survey was not anonymous with students being interviewed directly by the researcher and the questionnaires were provided only once.

In conclusion, we can emphasise the importance of promoting healthy lifestyle habits among university students and to begin this education from an early age, particularly with regards to the responsible alcohol consumption. Prevention strategies should be targeted from early childhood to university age and should be accompanied by strategies for the early detection of those at risk. Universities might pay attention to their alcohol policies with programs that could involve motivation to change alcohol misuse. Specific intervention health programs should be preventive to help into negative consequences of heavy episodes of drinking and hazardous alcohol use among higher education students.

Therefore, longitudinal studies are necessary to observe the evolution of these cognitive deficits and evaluate their consequences in the future, due to the permissiveness that some societies show regarding young alcohol consumption.

## Declarations

### Author contribution statement

López-Moreno Miguel, Garcés-Rimón Marta, Miguel Marta, Iglesias-López María Teresa: Conceived and designed the experiments; Performed the experiments; Analyzed and interpreted the data; Contributed reagents, materials, analysis tools or data; Wrote the paper.

### Funding statement

This research did not receive any specific grant from funding agencies in the public, commercial, or not-for-profit sectors.

### Data availability statement

Data will be made available on request.

### Declaration of interests statement

The authors declare no conflict of interest.

### Additional information

No additional information is available for this paper.
